# Eczema as a Danger Signal

**Published:** 1907-07

**Authors:** 


### ECZEMA AS A DANGER SIGNAL.

Bulkley reminds us that while eczema is so often regarded and too frequently treated as a wholly local disease of the skin, often without success, in certain cases it gives signals of danger of no uncertain character. These if heeded results to the best interests of the patient and the cure of the disease, but when, if disregarded, may result most disastrously.

Generalized eczema, or that affecting many localities, is almost always a sign of nervous or physical breakdown, and a most careful study of the patient in every particular will often reveal gross errors in life or habits, which, if unchecked, will lead to direful results. Localized neurotic eczema of the hands, or about the mouth, will also often indicate a nervous strain to which the patient may otherwise succumb unless properly attended to. Eczema about the eyes is sometimes due to eyestrain or errors of accommodation which call for hysterical attention.

But eczema is also not infrequently met with in those of full, plethoric habit, with a hard, bounding pulse, showing metabolic errors which may lead to kidney disease or even apoplexy if not controlled by proper measures. Again, many cases exhibit a weak, flabby pulse, indicating anemia or a greatly debilitated system, ready to succumb to disease, unless restored to a normal vigor by appropriate treatment.—Jour. A. M. A.

